# Validation of the relationship between coagulopathy and localization of hydroxyethyl starch on the vascular endothelium in a rat hemodilution model

**DOI:** 10.1038/s41598-021-89889-8

**Published:** 2021-05-21

**Authors:** Ryu Azumaguchi, Yasuyuki Tokinaga, Satoshi Kazuma, Motonobu Kimizuka, Kosuke Hamada, Tomoe Sato, Michiaki Yamakage

**Affiliations:** Department of Anesthesiology, Sapporo Medical University School of Medicine, South 1, West 16, Chuo-ku, Sapporo, Hokkaido 060-8543 Japan

**Keywords:** Experimental models of disease, Drug development, Adverse effects

## Abstract

Various anticoagulant properties have been associated with hydroxyethyl starch (HES). However, the mechanism remains unclear and it has not been fully considered whether these properties are beyond the dilutional effect itself. The aim of this study was to reproduce the coagulopathy induced by HES and to test the hypothesis that the coagulopathy is caused by endothelial or glycocalyx damage due to localization of HES on the endothelium, which is caused by the high shear viscosity of dilutional blood. Using a rat model, we compared blood coagulability measured by Sonoclot, levels of endothelial and glycocalyx damage markers and coagulation factors, and blood shear viscosity when hemodilution was performed with physiological saline (PS), 6% HES 130/0.4 in PS, and 10% HES 200/0.5 in PS. We also evaluated the localization rates of fluorescently labeled HES on endothelium in the isolated aorta. HES decreased the fibrin gel formation rate more than did PS. HES was shown to cover the endothelium, possibly due to its high shear viscosity, and this mechanism potentially acted to protect, rather than damage, the endothelium and glycocalyx. However, this covering effect may be the cause of coagulopathy due to inhibition of von Willebrand factor secretion from the endothelium.

## Introduction

Hydroxyethyl starch (HES) is believed to have a greater inhibitory effect on blood coagulation than physiological saline (PS)^1^. It has been reported that in vivo hemodilution with HES resulted in increased perioperative blood loss^2^, and that in vitro or in vivo hemodilution caused coagulopathy measured by point-of-care devices such as ROTEM, TEG, and Sonoclot^3^ as well as reductions in von Willebrand factor (VWF) antigen (Ag) levels and coagulation factor VIII (FVIII) Ag levels^4^. It has also been reported that the coating of platelets with HES molecules may inhibit contact of glycoprotein (GP) IIb/IIIa or GP Ib and coagulation factors on the platelet membrane^5^. However, the underlying mechanism integrating these phenomena is unknown. Furthermore, most of these studies did not fully satisfy isodilution, standardization of invasion, and in vivo metabolism of HES at the same time.

The vascular endothelium (ET) and glycocalyx (GCX) are known to play a role in controlling blood coagulation by displaying thrombomodulin (TM) and heparan sulfate proteoglycan (HSPG) and by secreting VWF^6^. Recent studies on sepsis and trauma have focused on damage to the ET and GCX as a mechanism of coagulopathy^7,8^. Septic and traumatic inflammatory reactions are known to damage and shed TM on the ET, and HSPG in the GCX^7,8^. Soluble TM in the blood activates protein C (PC), and activated PC (aPC) neutralizes coagulation factors V (FV) and FVIII. Eluted HSPG, which has the same function as heparin, acts as an endogenous anticoagulant substance by binding to antithrombin and inhibiting the activity of thrombin and FX. Dextran is a biopolymer of glucose produced by enzymes of certain bacteria and a colloid of molecular weight similar to that of HES. It is infused as a solute preparation to expand circulation plasma volume because of its high residual effect in vessels. In a classic animal study, radiocarbon-labeled dextran molecules were shown to have ‘adhered’ to the vascular ET via a non-covalent bond, which led to the conclusion that dextran is involved in suppressing the hemostatic process by blocking contact between coagulation factors and the ET^9^.

Recent studies have shown that HES is more damaging to the endothelium than albumin, and that crystalloid is more damaging than HES^10,11^. However, a subgroup analysis in a clinical trial reported that HES produced a greater reduction in coagulability compared with albumin by ROTEM, yet the mechanism was shown to be unrelated to ET or GCX damage^12^. The precise mechanism explaining the action of HES on the ET and GCX is currently unknown. The relationship between the anticoagulant effects of HES and damage to the ET and GCX has also not been established.

Therefore, in an attempt to clarify the mechanisms underlying the anticoagulant effects of HES, we reproduced coagulopathy induced by HES using a rat hemodilution model in which the hemodilution inequality was strictly eliminated. We hypothesized that HES causes coagulopathy by localization and impairment of the ET and GCX, and that its high shear viscosity due to non-covalent binding is the cause of HES localization because of the inherently immobilizing nature of high viscosity material.

## Methods

### Study animals

The Institutional Animal Care and Use Committee of Sapporo Medical University granted approval for the study (Permit Number: 16-095). All animal experiments were conducted during 2017–2019 in accordance with the ARRIVE guidelines. All experimental procedures were performed in accordance with the relevant guidelines and regulations. Male Wistar rats weighing 280–320 g were purchased from Japan SLC (Shizuoka, Japan) and kept in temperature (22–24 °C) and humidity (45–55%) controlled rooms with a 24-h light–dark cycle, and given standard chow and tap water ad libitum. All surgical procedures were performed under anesthesia, and every effort was made to minimize suffering.

### Determination of sample size

Sample size was determined using G*Power version 3.1.9.2 for analysis of the preliminary data for experimental hemodilution in which Sonoclot was used, and focused on measurement of the clot rate (CR). The level of statistical significance was set at 5%, the power was set at 80% and the number of groups was set at four. The calculated effect size was 0.57, and the total sample size needed for each experiment was calculated to be 40.

### Allocation of experimental animals

A total of 160 rats were used, 120 of which were divided into three groups of 40 rats each. The three groups were assigned as experimental groups for coagulability measurement, quantification of ET and GCX damage markers and coagulation factors, and shear viscosity measurement. In each experimental group, the 40 rats were further divided into four groups of 10, of which one group was assigned to the no-dilution group (control) and three hemodilution groups were assigned as follows: (1) PS, (2) 6% HES 130/0.4 in PS (6% Voluven; Fresenius Kabi AG, Bad Homburg, Germany) (HES130), and (3) 10% HES 200/0.5 in PS (10% Pentaspan; Jeil Pharm., Seoul, Korea) (HES200). The remaining 40/160 rats were used for quantitative optical evaluation of fluorescently labeled HES in the isolated aorta. All allocations were randomized.

### Experimental protocol for preliminary hemodilution

Rats were tracheostomized under inhalational anesthesia with 5% sevoflurane and 1 L/min 100% oxygen during spontaneous breathing. A venous catheter was inserted percutaneously in the tail vein and an arterial catheter was inserted in the femoral artery by the open approach under microscopic visualization. Subsequent procedures were performed under mechanical ventilation (2% sevoflurane) with continuous blood pressure monitoring. Body temperature was kept at 37 °C using an experimental animal heater (BWT-100A; Bio Research Center, Nagoya, Japan).

Each infusion preparation was administered at 1 ml/h in the vein and 3 ml/h in the artery. Anticoagulant agents were not used in order to exclude their potential effects on coagulability, shear viscosity of blood, and ET and GCX markers. Intra-catheter blood clotting was avoided by maintaining arterial infusion at the minimum rate. Arterial blood sampling was performed every 20 min and included 0.2 ml samples for blood gas analysis (EPOC; Epocal, Ottawa, Ontario, Canada) and 0.8 ml samples for hemodilutional blood removal. The 0.8 ml blood sampling was always performed immediately and continuously after the 0.2 ml blood gas analysis to ensure that the hematocrit (Ht) values would be the same in each. We considered that the accuracy of the blood gas analysis was guaranteed if the first Ht value was 34% or more. Blood aspiration was performed gently, to avoid unnecessary platelet activation. Each blood sampling and blood removal was followed by a 1 ml bolus infusion of the respective infusion preparation for volume compensation and arterial catheter flushing. An additional 0.5 ml intravenous administration for each infusion preparation was permitted without limitation to maintain a stable blood pressure and to avoid decreases of more than 20% in mean blood pressure. These infusion and aspiration procedures resulted in gradual in vivo hemodilution without clinical evidence of shock.

**Figure 1:**
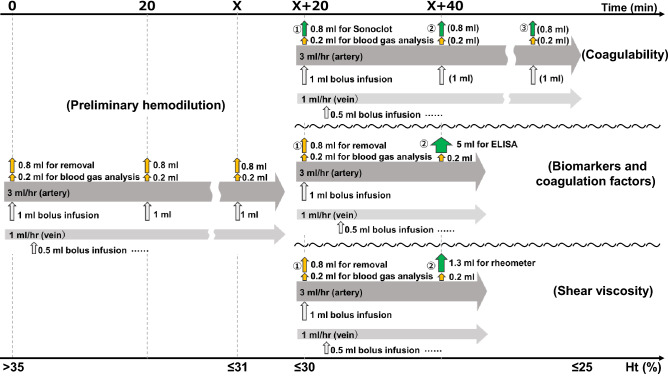
Hemodilution protocol. All hemodilution procedures were performed consecutively, first by the preliminary hemodilution method, followed by secondary hemodilution in different ways depending on what was measured. *Ht* hematocrit, *ELISA* enzyme-linked immunosorbent assay. *X* time at which the Ht level reached ≤ 31%. Yellow arrow, external; green arrow, external (sampling); white arrow, internal.

Serial hemodilution was initiated according to the procedures described above until the Ht level was reduced to 31% or less (Fig. 1). These procedures were defined as preliminary hemodilution. Fluid balance per body weight and the time required to achieve preliminary hemodilution were recorded. Following the preliminary hemodilution, hemodilution was continued for coagulability measurement, quantification of ET and GCX damage markers and coagulation factors, and shear viscosity measurement.

### Coagulability measurement using Sonoclot

Sonoclot (Sonoclot II Coagulation and Platelet Function Analyzer; Sienco, Morrison, CO, USA) is a point-of-care device for evaluation of whole blood clotting ability. The required blood sampling volume is 0.4 ml per channel, with a total of 0.8 ml for the two channels in the device. A vertically vibrating probe with a frequency of 200 Hz detects whole blood coagulation resistance that is output as a resistance curve, after activation by celite prefilled in a disposable cuvette. Activated clotting time (ACT) was defined as the time to start of blood clotting and represents the length of the horizontal part of the curve prior to the upslope of coagulation resistance. CR was defined as the rate of fibrin gel formation from fibrinogen, and represents the upslope angle of the coagulation resistance curve. Platelet function (PF) was defined as the strength of clot retraction. Sonoclot was selected for use based on previous studies that demonstrated its effective detection of coagulopathy induced by hemodilution with crystalloids and colloids in vitro^13^ and in vivo^14^.

### Quantification of ET and GCX damage markers and coagulation factors by ELISA

Commercially available ELISA kits were used for quantification. All assays were analyzed in duplicate. TM Ag (E-EL-R0960; Elabscience Biotechnology, Wuhan, China) and aPC Ag (SEA738Ra; Cloud-Clone, Katy, TX) were assayed to evaluate the extent of vascular ET damage by HES. Syndecan-1 (SDC-1) Ag (E-EL-R0996; Elabscience Biotechnology), which is known as a component of GCX and elutes into the blood when GCX is impaired, and HSPG Ag (E-EL-R0491; Elabscience Biotechnology) were assayed to evaluate the extent of GCX damage by HES. VWF Ag (E-EL-R1079; Elabscience Biotechnology), FV Ag (E-EL-R0230; Elabscience Biotechnology) and FVIII Ag (MBS749830; MyBioSource, San Diego, CA) from plasma were quantified to investigate the influence of these coagulation factors on the CR measured by Sonoclot. The whole blood volume required for all ELISA tests was calculated to be 5 ml, including storage, for each rat.

### Shear viscosity measurement using a rheometer

The shear viscosity of whole blood was measured using a rheometer (MCR102, CP50-1; Anton Paar, Graz, Austria). Recordings were performed when the shear rate ranged from 1 to 1000 s^−1^ at a temperature of 37 °C. The shear viscosity at a shear rate of 398 s^−1^ was adopted and compared between the no-dilution group and each of the three dilution groups. This shear rate has previously been estimated to be close to that of the rat femoral artery and descending aorta^15^. Each 1.3 ml blood sample obtained was added to a collecting tube prefilled with ethylenediaminetetraacetic acid to prevent clotting, and all of it was used for the measurement.

### Secondary hemodilution and sampling protocol following preliminary hemodilution

Following preliminary hemodilution, secondary hemodilution was continued in a different way for each of the three measurements until the Ht level was reduced to the range of 26–30% (Fig. 1). This hemodilution target was set prospectively because it is considered to approximate the conditions commonly found in clinical practice. All results were collected when the Ht levels were in the range of 26–30% except in the no-dilution group. To minimize invasiveness, blood samples for measurements and quantifications in the no-dilution group were collected just after arterial cannulation, without tracheostomy.

Coagulability measurements after hemodilution in which the Ht level was 31% or less were performed using 0.2 ml blood samples for blood gas analysis, 0.8 ml blood samples for Sonoclot measurement, and 1 ml arterial bolus infusion every 20 min during a 1 ml/h venous infusion and 3 ml/h arterial infusion. Additional 0.5 ml infusions were performed a few times. The blood samples for blood gas analysis and Sonoclot measurement were collected simultaneously. One to three Sonoclot measurements were recorded when the Ht level was 26–30%. Values of CR, ACT and PF were averaged in each rat for comparison between the no-dilution group and each of the three hemodilution groups.

Quantification of ET and GCX damage markers and coagulation factors and the shear viscosity measurements were performed continuously by the preliminary hemodilution protocol. Blood samples for these measurements and quantifications were collected at the time of the second 0.2 ml blood gas analysis rather than at the 0.8 ml removal because the Ht levels were expected to be in the target range of 26–30% at this time. These blood samples were collected only once in each rat because the amount of blood required for these examinations was too large to be drawn more than twice. All blood samplings were matched in the range of 26–30% Ht using this protocol.

### Quantitative optical evaluation of fluorescently labeled HES in the isolated aorta

The 40 rats were divided into four groups of 10 rats in each group. Fluorescein isothiocyanate (FITC) labeled 6% HES 130/0.4 in PS (FITC-HES130) was infused in the isolated aorta of one group; FITC labeled 10% HES 200/0.5 in PS (FITC-HES200) was infused in one group; and in two control groups, fluorescein sodium salt (uranine; 213-00092, FUJIFILM Wako Pure Chemical, Osaka, Japan) and either HES130 or HES200 was infused. FITC-HES130 and FITC-HES200 were prepared by mixing HES 130/0.4 powder (Fresenius Kabi AG, Bad Homburg, Germany), HES 200/0.5 powder (Carbosynth, Berkshire, UK), and the provided HES130 and HES200 powders which were adjusted to have a fluorescence modification rate of 25–37% per molecule for each (Peptide Institute, Osaka, Japan). The amount of pigment contained in all infusion preparations was adjusted to 6.0 × 10^−5^ mol/L.

Rats were anesthetized by inhalation of 5% sevoflurane and were sacrificed under narcosis by common carotid artery resection. After thoracotomy, the descending aorta was isolated immediately and bathed in 37 °C phosphate buffered saline (PBS). The aorta was prepared in 12 mm sections under microscopic visualization. An 18-gauge venous catheter (Supercath 5, Medikit, Tokyo, Japan) was cut in the mid-portion. The hub-side was inserted at the proximal end of the aorta and the tip was inserted at the distal end. Both sides were ligated with 3–0 silk thread. Infusion into the isolated aorta was performed with care to prevent the aorta from being unnecessarily bent or pulled. Each infusion preparation was infused at 5 ml/h for 20 min. For flushing, PBS was infused at 5 ml/h for 20 min followed by a 5 ml bolus infusion for 25 s.

**Figure 2:**
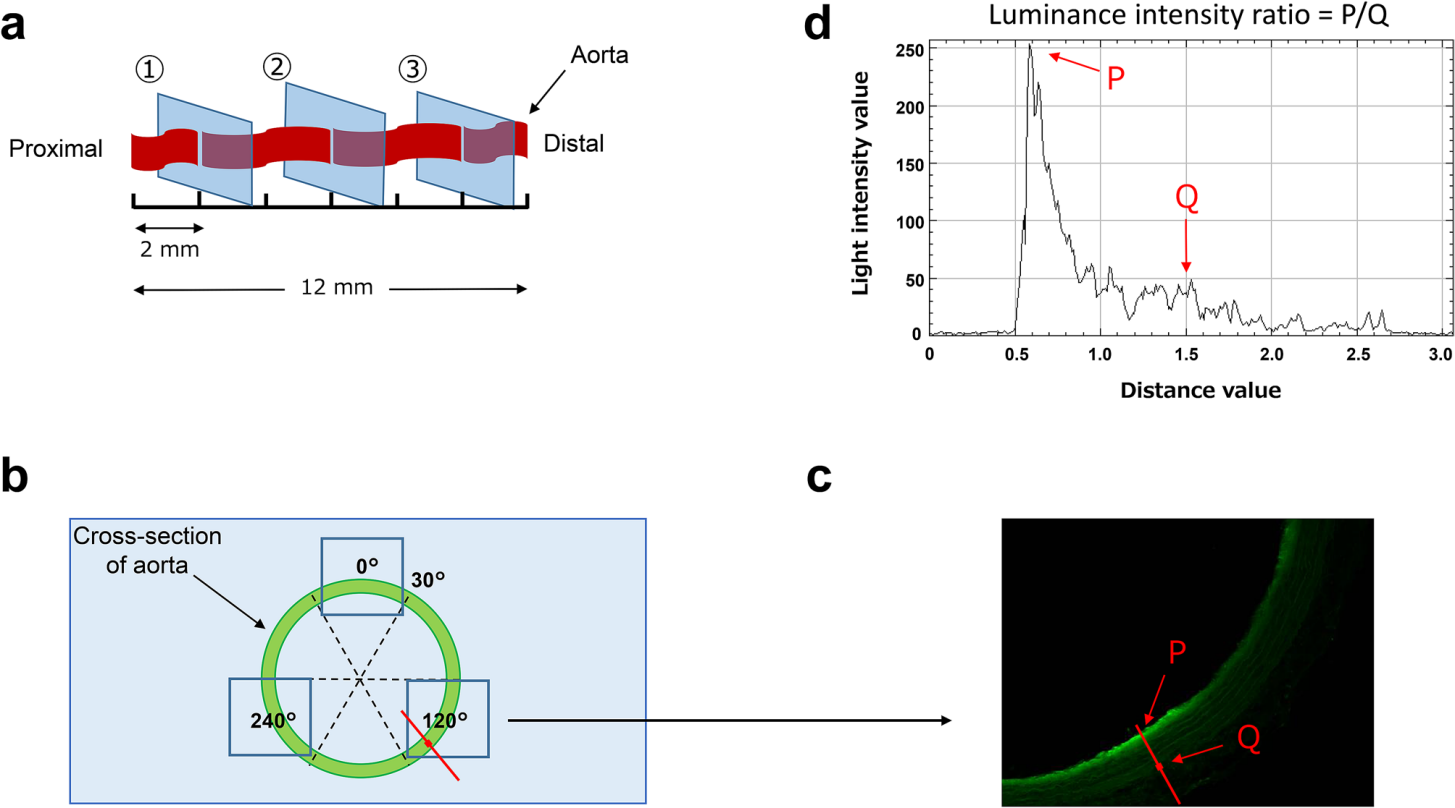
Evaluation of fluorescently labeled HES localized in the isolated aorta. (**a**) Collection of cross-sections from the isolated aorta. (**b**) Three images (squares) were taken in one cross-section divided into 6 parts. (**c**) A normal line was drawn from the point of highest luminance intensity on the endothelium (P) to the outer membrane (Q). (**d**) Endothelial luminance intensity ratio (P/Q).

After infusion, all aortas were immediately frozen and sectioned on a cryostat (CM3050 S; Leica, Nussloch, Germany) at a thickness of 40 μm. Three cross-sections were collected, at distances of 2, 6, and 10 mm from the proximal end of the aorta (Fig. 2a), and each was placed on a slide glass in a random orientation. The cross-sections were evaluated by a confocal laser scanning microscope (LSM 510 META; ZEISS, Jena, Germany). One cross-section was divided into 6 parts at 60° intervals from the 30° position, with 0° as the 12 o’clock position. Images of the 0°, 120°, and 240° sections were taken (Fig. 2b). Image J public domain software (NIH Image, Bethesda, MD, USA) was used for image analysis. In each image, a line was drawn normal to the point on the ET where the luminance intensity was the highest (P), and the middle point of this line was set on the outer membrane (Q) (Fig. 2c). On the normal line, the ratio of the ET luminance intensity was determined when the outer membrane was set as a reference (P/Q), to offset subtle differences in image shooting conditions (Fig. 2d). Nine ET luminance intensity ratios were calculated per aorta and these results were averaged. Luminance intensity was compared among the four groups. All image analysis was performed by the same person, who was blinded to information regarding the image shooting conditions.

### Statistical analysis

Statistical analyses were performed using Graph Pad Prism 7 (GraphPad Software, San Diego, CA, USA). Data distribution was assessed using the Shapiro–Wilk test. Data are presented as the mean (standard deviation) for normally distributed data or as the median (interquartile range) for other data. Statistical comparisons of normally distributed data were performed by one-way ANOVA followed by Tukey’s test for multiple comparisons. Statistical comparisons of other data were performed by Kruskal–Wallis test followed by Dunn’s test for multiple comparisons. P values < 0.05 were considered to be statistically significant.

## Results

### Hemodilution

Final Ht dilution levels were 27–28% in all hemodilution groups, and were significantly lower compared with the no-dilution group for each of the three measurements of coagulability, coagulation factors and endothelial and glycocalyx damage markers, and shear viscosity of blood (P < 0.0001) (Table 1). The fluid balance per body weight needed for common preparatory hemodilution was significantly lower in all HES groups compared with the PS group (P < 0.0001) (Table 1). There were no significant differences in the time needed for targeted dilution.

**Table 1:** Performance of hemodilution.

	No dilution	PS	HES130	HES200	*P*
**Preliminary hemodilution**					
n	30	30	30	30	
Fluid balance per body weight (× 10^−3^ ml/g)		35 (13)	13 (3.1)^§^	11 (6.2)^§^	< 0.0001
Time needed for targeted dilution (min)		102 (28)	88 (32)	84 (29)	0.4
**Hemodilution for the coagulability measurement**					
n	10	10	10	10	
Ht (%)	37 (1.4)	28 (0.93)*	28 (0.81)*	28 (1.1)*	< 0.0001
**Hemodilution for quantification of endothelial and glycocalyx damage markers and coagulation factors**					
n	10	10	10	10	
Ht (%)	36 (1.4)	29 (0.95)*	28 (1.1)*	28 (1.1)*	< 0.0001
**Hemodilution for the shear viscosity measurement**					
n	10	10	10	10	
Ht (%)	38 (1.7)	28 (1.4)*	28 (0.88)*	27 (1.1)*	< 0.0001

Data are presented as the mean (SD).

*Ht* hematocrit, *PS* physiological saline, *HES 130* 6% hydroxyethyl starch 130/0.4 in PS (6% Voluven), *HES 200* 10% hydroxyethyl starch 200/0.5 in PS (10% Pentaspan).

*P < 0.0001 vs no-dilution, ^§^P < 0.0001 vs PS.

### Coagulability measurement using Sonoclot

**Figure 3:**
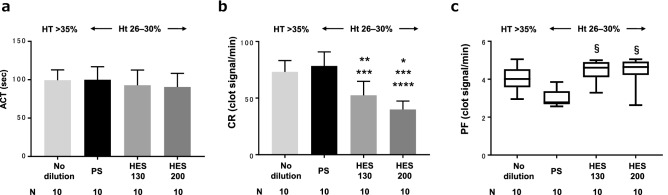
Coagulability measurement using Sonoclot. *ACT* activated clotting time, *CR* clot rate, *PF* platelet function. PS, physiological saline; HES 130, 6% hydroxyethyl starch 130/0.4 in PS (6% Voluven); HES 200, 10% hydroxyethyl starch 200/0.5 in PS (10% Pentaspan); Ht, hematocrit. *P < 0.0001 vs no-dilution, **P = 0.0007 vs no-dilution, ***P < 0.0001 vs PS, ****P = 0.047 vs HES130, ^§^P = 0.002 vs PS.

There was no significant difference in ACT values among the groups (Fig. 3a). CR values in the no-dilution, PS, HES130, and HES200 groups were 73 (10), 78 (12), 52 (13), and 40 (7.4) clot signal/min, respectively. There was a statistically significant difference between all pairs except between the no-dilution and PS groups (Fig. 3b). PF values were significantly higher in the HES130 group (4.6 [4.1, 4.9] clot signal/min) and the HES200 group (4.6 [4.2, 4.9] clot signal/min) compared with the PS group (2.8 [2.7, 3.4] clot signal/min) (P = 0.002) (Fig. 3c).

### Quantification of ET and GCX damage markers by ELISA

**Figure 4:**
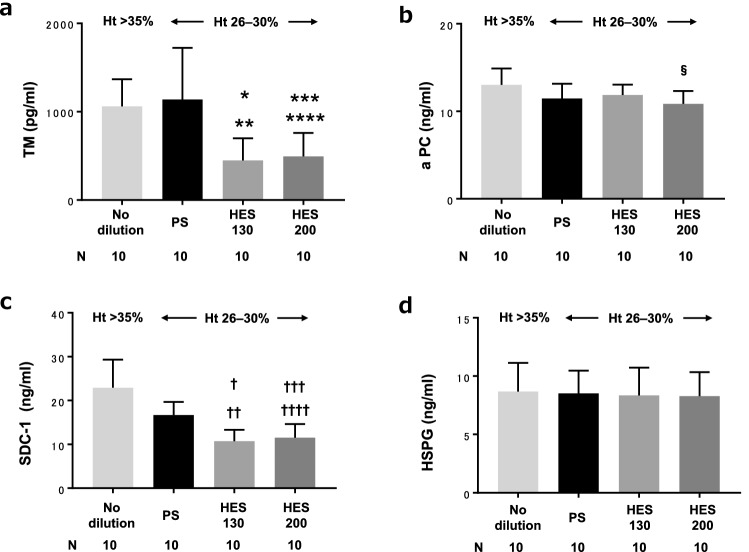
Quantification of Quantification of ET and GCX damage markers by ELISA. ET, endothelium; *GCX*, glycocalyx; *ELISA*, enzyme-linked immunosorbent assay; *TM*, thrombomodulin;*aPC*, activated protein C; *SDC*-1, syndecan-1; *HSPG*, heparan sulfate proteoglycan. *P = 0.008 vs no-dilution, **P = 0.003 vs PS, ***P = 0.016 vs no-dilution, ****P =0.005 vs PS, ^§^P= 0.021 vs no-dilution, ^†^P = 0.0009 vs no-dilution, ^††^P = 0.018 vs PS,^†††^P = 0.003 vs no-dilution, ^††††^P = 0.045 vs PS.

TM Ag levels for the HES130 group (450 [250] pg/ml) and HES200 group (490 [270] pg/ml) were significantly lower compared with the no-dilution group (1000 [310] pg/ml) (P = 0.008, P = 0.016, respectively) and PS group (1100 [580] pg/ml) (P = 0.003, P = 0.005, respectively) (Fig. 4a). APC Ag levels for the HES200 group (11 [1.5] ng/ml) were significantly lower compared with the no-dilution group (13 [1.9] ng/ml) (P = 0.021). No significant difference was found among any other pairs (Fig. 4b). SDC-1 showed a very similar trend to TM (Fig. 4c). There was no significant difference in HSPG Ag levels among the groups (P = 0.98) (Fig. 4d).

### Quantification of coagulation factors by ELISA

**Figure 5:**
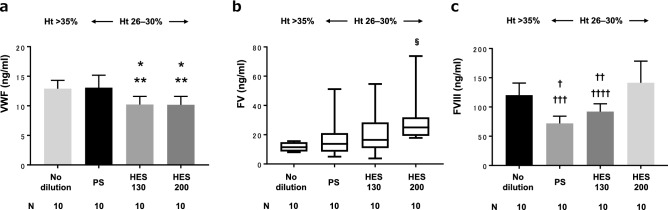
Quantification of coagulation factors by ELISA. *ELISA* enzyme-linked immunosorbent assay, *VWF* von Willebrand factor, *FV* coagulation factor V, *FVIII* coagulation factor VIII. *P = 0.003 vs no-dilution, **P = 0.001 vs PS, ^§^P = 0.03 vs no-dilution, ^†^P < 0.0001 vs no-dilution, ^††^P = 0.004 vs no-dilution, ^†††^P < 0.0001 vs HES200, ^††††^P = 0.0002 vs HES200.

VWF Ag levels for the HES130 and HES200 groups (each 10 [1.4] ng/ml) were significantly lower compared with the no-dilution group (13 [1.4] ng/ml) (P = 0.003) and PS group (13 [2.1] ng/ml) (P = 0.001) (Fig. 5a). FV Ag levels were significantly higher in the HES200 group (25 [19, 32] ng/ml) compared with the no-dilution group (11 [8.6, 15] ng/ml) (P = 0.03). No significant difference was found among any other pairs (Fig. 5b). FVIII Ag levels for the PS group (72 [12] ng/ml) and the HES130 group (92 [13] ng/ml) were significantly lower compared with the no-dilution group (120 [21] ng/ml) (P < 0.001, P = 0.004, respectively) and the HES200 group (140 [37] ng/ml) (P < 0.001, P = 0.002, respectively) (Fig. 5c).

### Shear viscosity measurement by rheometer

**Figure 6:**
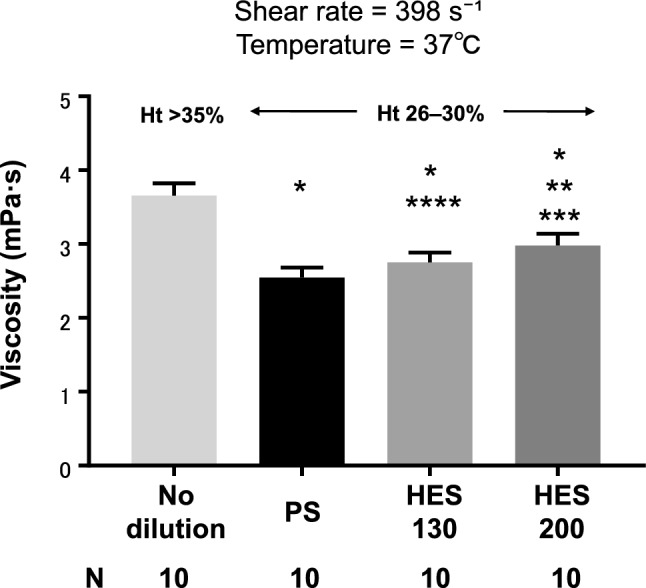
Shear viscosity measurement by rheometer. *PS* physiological saline, *HES130* 6% hydroxyethyl starch 130/0.4 in PS (6% Voluven), *HES200* 10% hydroxyethyl starch 200/0.5 in PS (10% Pentaspan). *P < 0.0001 vs no-dilution, **P < 0.0001 vs PS, ***P = 0.01 vs HES130, ****P = 0.032 vs PS.

Shear viscosity of blood was significantly different among all the hemodilution groups. Values of shear viscosity in the no-dilution, PS, HES130, and HES200 groups were 3.7 (0.17), 2.5 (0.13), 2.8 (0.13), and 3.0 (0.16) (mPa s), respectively (Fig. 6).

### Localization of fluorescently labeled HES in the vascular ET of the isolated aorta

**Figure 7:**
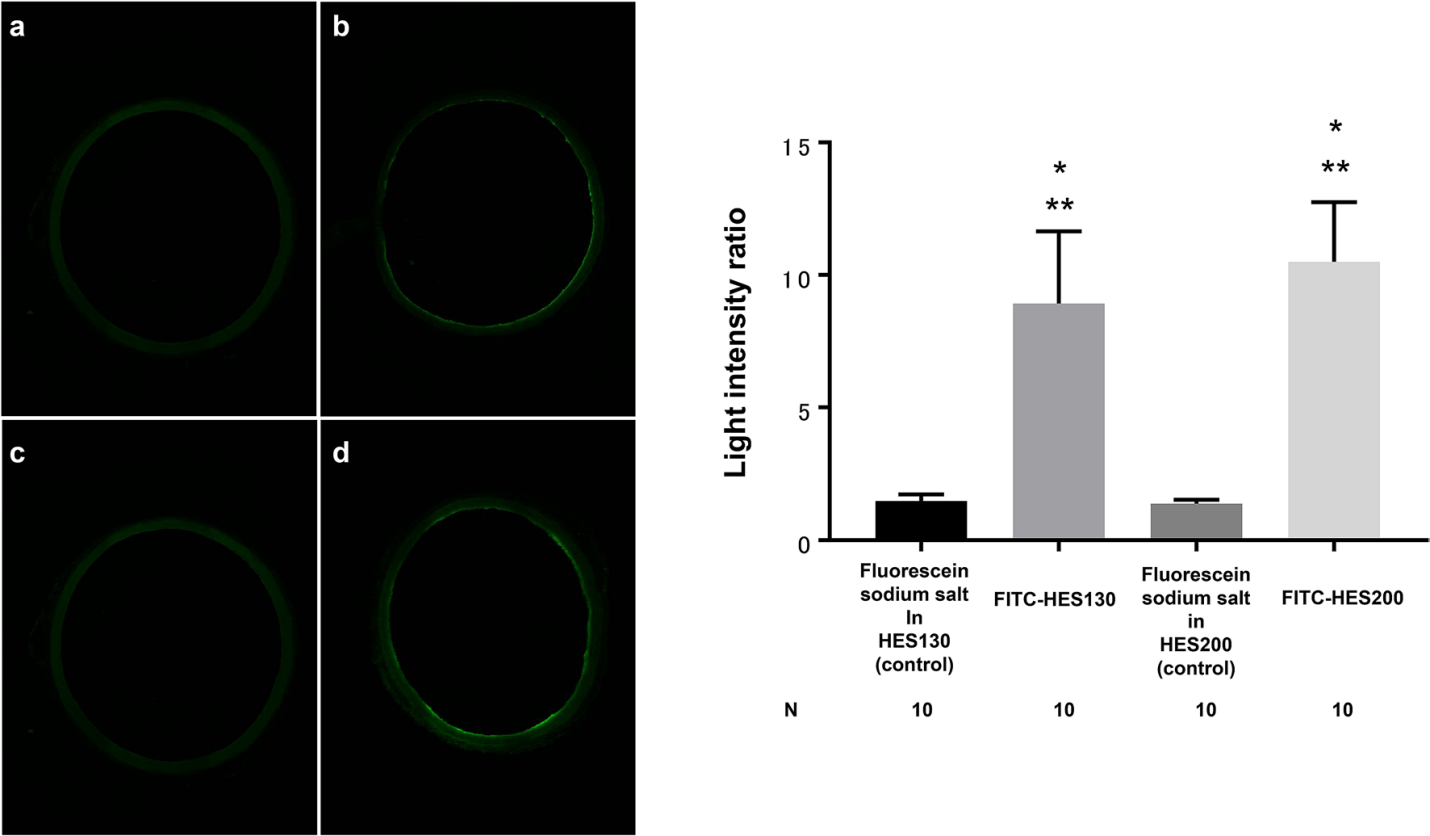
Left: Representative cross-sectional images of the isolated aorta after infusion of fluorescently labeled HES. (**a**) Fluorescein sodium salt in HES130 (control), (**b**) FITC-HES130, (**c**) fluorescein sodium salt in HES200 (control), (**d**) FITC-HES200. Right: Luminance intensity ratio of endothelium to outer membrane after infusion of fluorescently labeled HES in the isolated aorta. *HES130* 6% hydroxyethyl starch 130/0.4 in PS (6% Voluven), *HES200* 10% hydroxyethyl starch 200/0.5 in PS (10% Pentaspan), *FITC* fluorescein isothiocyanate. *P < 0.0001 vs fluorescein sodium salt in HES130 (control), **P < 0.0001 vs fluorescein sodium salt in HES200 (control).

Cross-sectional images show localization of FITC-HES130 and FITC-HES200 on the vascular ET (Fig. 7a). The light intensity ratio at the outer membrane of the ET was significantly higher in both the FITC-HES130 group (9.0 [2.7]) and FITC-HES200 group (10.5 [2.2]) compared with fluorescein sodium salt in the HES130 group (1.5 [0.25]) (P < 0.0001) and fluorescein sodium salt in the HES200 group (1.4 [0.15]) (P < 0.0001), respectively. There was no significant difference in light intensity ratio between the FITC-HES130 and FITC-HES200 groups (P = 0.49) (Fig. 7b).

## Discussion

This study compared blood coagulability, levels of ET and GCX damage markers and coagulation factors, and shear viscosity under conditions of hemodilution performed equally with PS, HES130, and HES200. Additionally, vascular ET localization of fluorescently labeled HES130 and HES200 was examined. The results showed that HES impaired coagulability; however, contrary to our hypothesis, HES acted more protectively on the ET and GCX compared with PS. HES resulted in increased shear viscosity and demonstrated localization to the vascular ET. There was a significant difference between HES130 and HES200 in terms of coagulability (Fig. 3b) and shear viscosity (Fig. 6). There was no significant difference between HES130 and HES200 in the quantification of ET and GCX damage markers (Fig. 4), coagulation factors (Fig. 5a), or vascular localization of HES, but HES200 tended to be localized to a greater extent than did HES130 (Fig. 7). Previous studies have demonstrated coating of HES around platelets^5^ and localization of dextran on the ET^9^. These results suggest that artificial colloids tend to ‘adhere’ to objects.

To specifically evaluate the basic mechanisms of coagulopathy induced by HES, we chose the following constructs for evaluation: (1) use of an in vivo model, (2) standardization of factors affecting coagulation (e.g., surgical invasion), and (3) dilutional equality taking into consideration the intravascular residual rates of the infusion preparations. These conditions were chosen because it was necessary to create a hemodilution model that maintained cell metabolism by α-amylase, avoided unnecessary activation of coagulation factors or platelets, and essentially eliminated the dilutional effect itself. Our hemodilution model met all of these conditions. Infusion of large volumes often causes hypothermia. We maintained the body temperature of the experimental animals at 37 °C because hypothermia reduces the activity of coagulation factors and acts to suppress coagulation. A recent study has reported that altered coagulation in specific-pathogen-free (SPF) animals was restored to normal host physiology on their return to a conventional facility^16^. To minimize the influence of SPF, in the present study we used original SPF rats that were bred in conventional environment.

The localizing and protective effects of HES on the ET both showed the same trend, which suggests that localization of HES on the ET may play a role in covering and protecting the ET and GCX. GCX functions primarily to regulate vascular permeability, and protection of GCX function would help to prevent leakage of the infused fluid^17^. This is consistent with the present result that the amount of infusion preparation used in the HES130 and HES200 groups was less than in the PS group (Table 1). In addition, previous studies have shown that volume overload causes ET and GCX damage by producing increases in atrial natriuretic peptide^18^. Likewise, increased fluid infusion has been correlated to GCX damage^19^. Therefore, the difference in the amount of infusion preparation itself could support the property that HES is protective to the ET and GCX. Despite dilution, Ag levels of TM and SDC-1 in the PS group were not significantly decreased compared with the no-dilution group, whereas those in the HES130 and HES200 groups were significantly decreased compared with the no-dilution group and the PS group (Fig. 4a,c). This result indicates that PS could potentially act to harm the ET and GCX, and induce shedding to increase TM and SDC-1 Ag levels, but that TM and SDC-1 shedding would be less for HES130 and HES200 than for PS. This means that HES acted more protectively on the ET and GCX compared with PS. We found that aPC Ag levels were lower for HES200 than for no-dilution (Fig. 4b). This is consistent with the finding of decreased TM and increased FV Ag levels for HES200 compared with no-dilution (Figs. 4a, 5b). Increased aPC Ag may be related to ET damage^11^. The decrease in aPC Ag could be explained by the dilutional effect and additional ET protection of HES200 in this situation. It is unclear why there was no difference in levels of HSPG Ag (Fig. 4d), which is a marker of GCX damage, as is SDC-1. It can be presumed that the morphological difference between HSPG and SDC-1^20^ could be responsible for differences in the level of shedding by hydrodynamic impact.

Interestingly, despite the protective action of HES on ET and GCX, HES impaired coagulability to a greater extent than did PS, as indicated by the decreased CR by Sonoclot and the decreased VWF Ag levels. The CR by Sonoclot mainly represents the degree of fibrin gel formation from fibrinogen. No studies have shown directly that a decrease in VWF Ag levels can be detected by Sonoclot. However, some previous studies have reported that TEG, which is also based on shear viscosity of blood measurement, can detect a decrease in both Ag levels and activity of VWF^21^. Sonoclot may also be capable of detecting decreases in VWF Ag levels because both tests monitor the process of whole blood clotting initiated by the addition of a coagulation activator. In our study, Sonoclot detected the coagulopathy induced by HES in spite of mild dilution. In contrast, a previous study showed that in vitro hemodilution with HES did not reduce tissue factor-stimulated fibrin thrombus formation as much as did saline at mild dilutions of 10–20%, which are similar to the dilution levels in the present experiment^22^. This may be for the reason that the action of HES on the ET exerts additional and practical influences on the coagulation system that cannot be observed in an in vitro study.

Decreases in VWF Ag levels showed a trend similar to that of the ET and GCX damage markers and a negative correlation with the covering rate of HES on the ET. VWF is produced in ET cells and is mostly secreted directly, with some stored in the Weibel–Palade bodies, which are responsible for regulating acute secretion of VWF^23^. If there is copious acute VWF secretion and secretion points are limited, total VWF Ag levels decrease even when little of the ET surface is covered and the half-life of VWF is moderately long. To verify whether covering of the ET and GCX by HES for less than one hour can reduce VWF levels, it is necessary to confirm an increasing rate of VWF secretion in vivo during the acute phase. Significant decreases in VWF Ag levels or activity have not been observed in vitro^24^, which also suggests that HES may inhibit VWF secretion from the ET.

PF measured by Sonoclot was significantly high in both HES groups compared with the PS groups (Fig. 3c). This result is inconsistent with that of a previous study^5^. PF reflects strength of clot retraction in which the distance between platelets is shortened through the binding of GIIb/IIIa and fibrinogen or GPIIb/IIIa and VWF during the blood clotting process^25^. A previous study reported that only HES130 did not decrease GPIIb/IIIa on platelets, in contrast to the effects of HES200, HES450, and HES70^26^. HES130 has been shown to activate immunological platelet function^27^. These findings agree with our results; however, the mechanism remains unknown. It is known that shear stress activates platelets^28^ and increases as shear viscosity increases. Although viscosity was highest in the no-dilution group, there were no significant differences in PF values between the other groups. This finding can be explained by the argument that due to high shear viscosity, HES did not cause a decrease in PF values, which were predicted to decrease to the same extent as for PS by the dilutional effect. This phenomenon may only be observed in cuvettes, where shear stress can be generated by vibration of the testing probe at 200 Hz.

FVIII Ag levels were lower for PS and HES130 compared with no-dilution, but paradoxically were higher for HES200 compared with PS and HES130 (Fig. 5c). It is known that FVIII binds to VWF and remains stable in the bloodstream^29^. Even in patients with acquired von Willebrand disease, FVIII decreases as VWF Ag decreases^30^. Recent studies have reported spontaneous increase of VWF Ag levels in FVIII-deficient mice^31^, decreased VWF Ag following administration of FVIII concentrate in patients with hemophilia^32^, and decreased FVIII activity in vitro in the presence of VWF Ag^33^. These findings suggest that FVIII and VWF might be in a state of equilibrium, and the possibility that a decrease in VWF can produce a surplus of FVIII. Further research on the processing of FVIII and VWF is required. A prior study has reported that Sonoclot was not able to detect increases in FVIII activity after administration of desmopressin^34^. The results of the present study indicate that the difference in FVIII Ag levels between HES200 and other infusion preparations may not have affected Sonoclot measurement. In addition, decreased aPC Ag supported higher levels of FVIII Ag in HES200.

There were several limitations in our study. We did not demonstrate a direct interaction among blood shear viscosity, vascular ET localization of HES, ET and GCX damage, and blood coagulability when the hemodilution was performed with HES. We quantified the Ag levels of coagulation factors but did not measure respective activity among them. The present result for aortic localization of HES may not be applied to the in vivo situation, for the reason that the vulnerability of GCX is unclear when it is not receiving blood flow or oxygen supply in vitro*,* although it is known that GCX is also distributed in the rat aorta and exists even after isolation^35^. FITC-HES was infused into the isolated aorta in a steady rather than pulsatile flow, and the infusion rate was slower than that of the real aorta. In addition, it can be assumed that in comparison to the isolated aorta, the capillaries occupy the majority of the blood volume in vivo, and there is greater diversity in combinations vessel diameter and blood flow velocity. Most of the infusion and blood sampling was arterial, which is different from actual clinical practice.

## Conclusions

Our study showed that hemodilution with HES was associated with localization of HES on the ET and covering of the ET and GCX, which was probably due to the high shear viscosity of HES. HES may act to protect rather than damage the ET and GCX. Covering of the ET by HES can limit VWF secretion from the ET and cause coagulopathy by reducing VWF Ag levels. These findings may help to comprehensively explain the various anticoagulant effects of HES reported so far and to clarify the underlying mechanism of the coagulopathy induced by HES.

## Data Availability

The data collected and analyzed in the current study are available from the corresponding author on reasonable request.
