# Author Correction: Validation of the relationship between coagulopathy and localization of hydroxyethyl starch on the vascular endothelium in a rat hemodilution model

**DOI:** 10.1038/s41598-021-97486-y

**Published:** 2021-08-31

**Authors:** Ryu Azumaguchi, Yasuyuki Tokinaga, Satoshi Kazuma, Motonobu Kimizuka, Kosuke Hamada, Tomoe Sato, Michiaki Yamakage

**Affiliations:** Department of Anesthesiology, Sapporo Medical University School of Medicine, South 1, West 16, Chuo-ku, Sapporo, Hokkaido 060-8543 Japan

Correction to: *Scientific Reports* 10.1038/s41598-021-89889-8, published online 21 May 2021

The original version of this Article contained an error in the legend of Figure 4.

“Quantification of ET and GCX damage markers by ELISA. *ET* endothelium, *GCX* glycocalyx, *ELISA* enzyme-linked immunosorbent assay, *TM* thrombomodulin, *aPC* activated protein C, *SDC-1* syndecan-1, *HSPG* heparan sulfate proteoglycan. *P = 0.008 vs no-dilution, **P = 0.003 vs PS, ***P = 0.016 vs no-dilution, ****P = 0.005 vs PS, ^§^P = 0.021 vs no-dilution, ^†^P = 0.0009 vs no-dilution 0.018 vs PS, ^††^P = 0.018 vs PS 0.0009 vs no-dilution, ^†††^P = 0.003 vs no-dilution, ^††††^P = 0.045 vs PS.”

now reads:

“Quantification of ET and GCX damage markers by ELISA. ET, endothelium; *GCX, glycocalyx*; *ELISA*, enzyme-linked immunosorbent assay; *TM*, thrombomodulin; *aPC*, activated protein *C*; *SDC-1*, syndecan-1; *HSPG*, heparan sulfate proteoglycan. *P = 0.008 vs no-dilution, **P = 0.003 vs PS, ***P = 0.016 vs no-dilution, ****P =0.005 vs PS, ^§^P = 0.021 vs no-dilution, ^†^P = 0.0009 vs no-dilution, ^††^P = 0.018 vs PS, ^†††^P = 0.003 vs no-dilution, ^††††^P = 0.045 vs PS.”

The original Article has been corrected.

